# Bilateral facial palsy caused by HIV infection: A case report and literature review

**DOI:** 10.1097/MD.0000000000033263

**Published:** 2023-03-17

**Authors:** Huiqian Lin, Haojie Hu

**Affiliations:** a Department of Neurology, Shijiazhuang People’s Hospital, Hebei, China; b Department of Neurology, Guiyang First People’s Hospital, Guizhou, China.

**Keywords:** autoimmune diseases, Bell palsy, bilateral facial palsy, HIV infection

## Abstract

Bilateral facial palsy (BFP) has been identified as a possible neurological complication of human immunodeficiency virus (HIV) infection, but only a limited number of cases have been reported in the literature. The purpose of this study was to deepen our understanding of the etiology of BFP. Case report: We report the case of a 46-year-old married bisexual man with BFP associated with HIV infection. The patient underwent serological testing for HIV and was positive. In the absence of any other evidence of underlying systemic disease, facial palsy is thought to be secondary to HIV infection. After antiretroviral therapy, the patient recovered completely from facial palsy within 3 months. Results: HIV infection often involves BFP. The pathophysiology of this clinical presentation is thought to be related to the immune response to the systemic transmission of the virus. Conclusions: Most patients with BFP have underlying systemic causes, particularly autoimmune diseases. The exclusion of HIV infection in patients with BFP is essential for the early diagnosis and management of HIV.

## 1. Introduction

As human immunodeficiency virus (HIV) infections increase, many opportunistic infections occur. Among them, nervous system complications are an important cause of morbidity and death in HIV-infected patients and can occur at any stage of infection involving any level of the central or peripheral nervous system.^[1]^ The complications include aseptic meningitis, meningoencephalitis, encephalitis, and peripheral nerve palsy. However, few studies have linked HIV infection with bilateral facial palsy (BFP).^[2]^ Facial palsy, also known as “Bell palsy,” is a common cranial neuropathy that can lead to paralysis of the facial muscles or complete paralysis on 1 side.^[3]^ The onset is sudden, and the duration of the disease can exceed 48 hours. It is primarily considered idiopathic and is diagnosed when other causes are excluded.^[3]^ BFP is extremely rare, accounting for only 0.3 to 2.0% of facial paralysis cases.^[4]^ Unlike unilateral facial palsy, it is usually caused by a serious systemic disease and, therefore, requires urgent medical intervention. Differential diagnosis is extensive, and a detailed history, physical examination, and investigation are necessary to determine the cause. Common causes include infection,^[5]^ autoimmune diseases,^[6]^ trauma,^[7]^ vasculitis,^[8]^ tumor,^[9]^ etc. BFP is characterized by bilateral nerve paralysis with symptoms occurring simultaneously or within 30 days. This case report describes a male patient who developed BFP due to exposure to cold. After imaging and laboratory tests, these symptoms were attributed to immunodeficiency due to the HIV infection.

## 2. Case report

**Figure 1.**
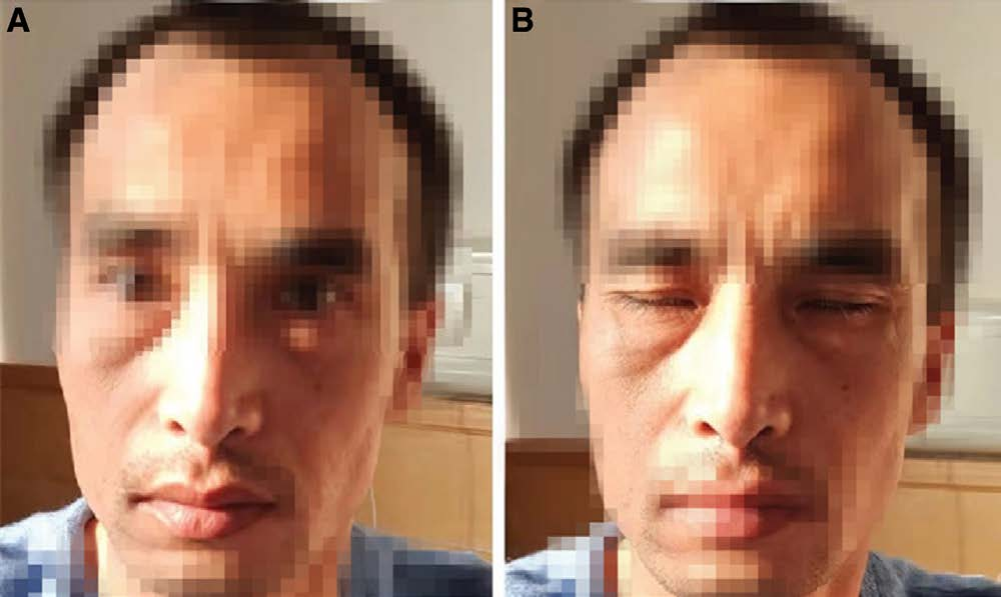
The patient was expressionless when (A) smiling and (B) attempting to close her eyes.

**Figure 2.**
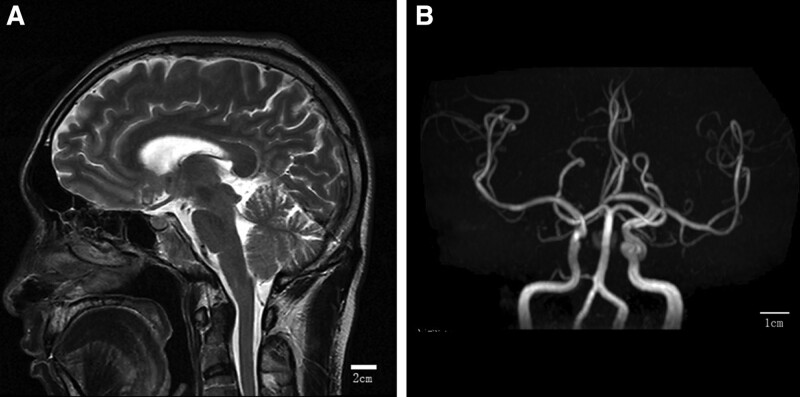
Imaging examination on admission showed no obvious abnormality in head MR. (A) MRI. (B) MRA. MRA = magnetic resonance angiography, MRI = magnetic resonance imaging.

A 46-year-old man with BFP was admitted to our hospital. The patient was admitted because of a sagging mouth, incomplete closure of both eyes, water leakage from both horns, pain in both ears, no herpes, no loss of taste, no limb movement disorder, and no limb numbness. No obvious abnormalities were found on computed tomography, and no obvious improvement was observed after anti-infection treatment. Symptoms begin with weakness on the right side of the face and progress to the left within 3 days. Deny any history of trauma, rash, travel abroad, or tick bite. On examination, the patient had bilateral facial muscle weakness, inability to raise eyebrows, puffy cheeks, smile without expression, and inability to close his eyes completely (Fig. 1). Neurological examination revealed bilateral inferior motor neuron paralysis with bilateral frontal striae lightening, hypophasis, and shallow nasolabial sulcus. The remaining neurological tests did not reveal any abnormalities. Blood cell analysis, hemagglutination, total biochemistry, thyroid function, tumor, cerebrospinal fluid (CSF) analysis, head computed tomography, and head magnetic resonance imaging revealed no significant abnormalities (Fig. 2). Epstein–Barr virus (EBV) antibody-negative, normal hepatitis series, syphilis-negative, HIV-positive, sent to the Center for Disease Control and Prevention for verification and confirmation of HIV antibody positivity. Supplementary diagnosis: HIV-positive. The patient had a history of homosexual sex. Diagnosis: Bilateral facial paralysis. Etiology: HIV infection. HIV was detected by enzyme-linked immunosorbent assay and western blotting, and 3 combinations (lamivudine, tenofovir, and efavirenz) were initiated for HIV neuropathy. After 14 days, she was discharged to the outpatient clinic for follow-up, and the facial paralysis disappeared within 3 months.

## 3. Discussion

BFP is very rare, affecting only 1 in 5 million people. Most cases of BFP are severe. Identifying the cause is key. A thorough blood test should be followed by detailed history and physical examination. In our case, the patient had a flu-like illness with fever, cough, and runny nose in the week prior to the onset. Finally, the patient was screened for HIV-positive acquired immunodeficiency syndrome (AIDS).

BFP mostly occurs in cases of Guillain-Barré syndrome (GBS),^[10]^ the cause of which is not yet clear and is generally believed to be an autoimmune disease. GBS is often accompanied by brain nerve damage, with BFP being common. Auxiliary examination shows protein-cell separation of the CSF, and there may be a delay or disappearance of the F wave or H reflex in the early stages of electrophysiological study.^[10]^ BFP has recently been reported to be associated with coronavirus disease 2019 vaccination.^[11]^ An increasing number of GBS cases have been reported among patients receiving coronavirus disease 2019 vaccination, both in the preclinical phase and after mass approval by authorities.^[12]^ The patient in our study was unvaccinated and could be excluded. This case was characterized by BFP. CSF analysis showed normal parameters and brain imaging showed no abnormalities. BFP develops rapidly, suggesting an infectious or autoimmune cause, particularly in the absence of a history of trauma or intracranial tumor. Lyme disease^[13]^ is also a common infectious cause of BFP, which is serologically negative and excluded. Our patient denied recent travel to endemic areas or exposure to ticks and was therefore considered unlikely. Acute EBV infection was excluded based on a negative EBV capsid antigen IgM antibody test.^[14]^ Other common systemic causes, including diabetes and normal blood sugar levels, can be ruled out.

BFP is a rare condition with a clear etiology that may be related to HIV infection. The pathophysiology of this clinical presentation is thought to be related to the immune response to the systemic transmission of the virus. AIDS is an infectious disease caused by HIV that attacks the immune system of the body. In people infected with HIV, 40 to 90% develop flu-like symptoms known as acute retrovirus syndrome (ARS), which is characterized by fever, myalgia, headache, rash, swollen lymph nodes, and neurological complications including aseptic meningitis, encephalopathy, neuropathy, myelopathy, and brachial neuritis. BFP caused by ARS generally appears approximately 15 days after the occurrence of ARS and has also been reported to be the first symptom of HIV infection.^[15]^ HIV infection should be suspected if peripheral facial palsies such as BFP, recent influenza-like illness, or Ramsay Hunt syndrome with disseminated shingles is present in high-risk populations. There have been reports of AIDS complicated by GBS,^[16]^ and the common pathogenesis is autoimmune disease. HIV is generally neurotropic and the location of the virus in the facial nerve or geniculate ganglion can cause edema and fibrous swelling within the nerve, resulting in enhanced signals in the affected facial nerve on brain magnetic resonance imaging. As facial palsy is considered the first clinical manifestation of HIV infection, it can aid in early diagnosis. As early treatment and prevention of HIV transmission are recommended, it is important to recognize the first signs of HIV infection; this should be considered a possible cause of facial palsy, especially in the younger population. Here, we describe a case of BFP followed by a diagnosis of HIV. This case highlights the importance of screening for HIV infection in patients with BFP.

## 4. Conclusion

The BFP, which is based on the immune system, is a specific form of Bell palsy. Clinicians should identify possible serious causes through thorough examinations to provide opportunities for early diagnosis and intervention in immune-related diseases.

## Author contributions

HQL wrote the initial draft and prepared the figures and submitted the article. HJH collected the literature and review the literature. The authors read and approved the final manuscript.
